# Preparation and Properties of Tumor-Targeting MRI Contrast Agent Based on Linear Polylysine Derivatives

**DOI:** 10.3390/molecules24081477

**Published:** 2019-04-15

**Authors:** Xuanrong Sun, Yue Cai, Zhuomin Xu, Dabu Zhu

**Affiliations:** Collaborative Innovation Center of Yangtze River Delta Region Green Pharmaceuticals, Zhejiang University of Technology, Hangzhou 310014, China; chinacaiyue@163.com (Y.C.); zhuominX9551@163.com (Z.X.); 15757116042@163.com (D.Z.)

**Keywords:** magnetic resonance imaging (MRI), polylysine, tumor targeting, charge reversal

## Abstract

We developed a tumor-targeted contrast agent based on linear polylysine (PLL) by conjugating a small molecular imaging agent, fluorescent molecule and targeting agent amino phenylboronic acid onto the amino groups of polylysine, which can specifically target monosaccharide sialic acid residues overexpressing on the surface of tumor cell membranes. Further, 3,4,5,6-Tetrahydrophthalic anhydride (DCA) was attached to the free amino groups of the polylysine to change to a negative charge at physiology pH to lower the cytotoxicity, but it soon regenerated to a positive charge again once reaching the acidic intratumoral environment and therefore increased cell uptake. Laser confocal microscopy images showed that most of the polymeric contrast agents were bound to the cancer cell membrane. Moreover, the tumor targeting contrast agent showed the same magnetic resonance imaging (MRI) contrasting performance in vitro as the small molecule contrast agent used in clinic, which made it a promising tumor-targeting polymeric contrast agent for cancer diagnosis.

## 1. Introduction

Cancer can be eradicated by chemotherapy, surgery and other methods in its early stages, but by the time it has metastasized, these methods almost never make a difference [1,2]. Therefore, accurate diagnosis of early tumors can greatly improve the survival rate and quality of life of patients [3]. Magnetic resonance imaging (MRI), as a safe, rapid and accurate clinical diagnosis method causing no harm to the human body, plays a wide role in tumor diagnosis and treatment [4,5].

Contrast agent (CA) is one of the most commonly used drugs in interventional radiology operations, which can effectively improve MRI sensitivity [6,7]. The application of small molecule gadolinium chelates in clinic has many disadvantages, such as nonspecificity, short survival time in vivo and low mobility [8]. In recent years, research on large molecule Gd chelates has become highly sought after. It has the advantages of high relaxation efficiency, low cytotoxicity and good tumor targeting [9], which can be better used in MRI tumor diagnosis [10,11]. However, the efficiency of macromolecular Gd chelates is still unsatisfactory. Only a small portion of CAs can reach the tumor site through the EPR effect, and most CAs remain in blood circulation or clear very quickly. Therefore, introducing some targeting groups to modify the CAs can achieve active binding to specific sites and enhance accumulation on tumor cell surface, which can further reduce toxicity for normal tissues and improve the contrast signals between the tumor and the surrounding tissues.

Tumor cells overexpressing sialic acid (Sia) appear protected against the immune defense system, and, as a result, malignancy is increased [12,13]. Numerous studies have demonstrated that sialic acid residues are relevant biomarkers of metastatic activity of tumors [14], and that the amount of Sia expression on cancer cells correlates with the prognosis of patients [15,16]. Phenylboronic acid (pba) moiety can recognize Sia on the cancer cell surface based on the reversible formation of five- and six-membered cyclic boronic acid/boronate esters between pha and the exocylic polyol function of Sia [17]. More importantly, phenylboronic acid/phenylboronate esters and their end products are considered nontoxic to humans [18,19,20]. Therefore, they are considered a potential candidate for active targeting the highly expressed Sia moiety in tumor cells.

Herein, polylysine (PLL) was used as a backbone, and we utilized a large number of amine groups in its side chain and afterwards linked with small molecule contrast agents, fluorescent dye and tumor-targeting groups boronic acid to obtain a novel polymeric contrast agent for active targeting of tumor cells. Meanwhile, the excess amino groups in PLL were treated by 3,4,5,6-tetrahydrophthalic anhydride (DCA) so that the whole contrast agent molecules exhibited a negative charge at physiological pH and resulted in less toxicity to the cells. Once reaching tumor sites, the anhydride in DCA quickly hydrolyzed and regenerated the original amine groups carrying cationic charge due to the tumor acidic environment [21,22,23], therefore significantly increasing the uptake of the contrast agent by the cancer cells and the selectively enhanced tumor imaging ability of the contrast agent.

## 2. Results and Discussion

### 2.1. Polymer Characterization

**Figure 1:**
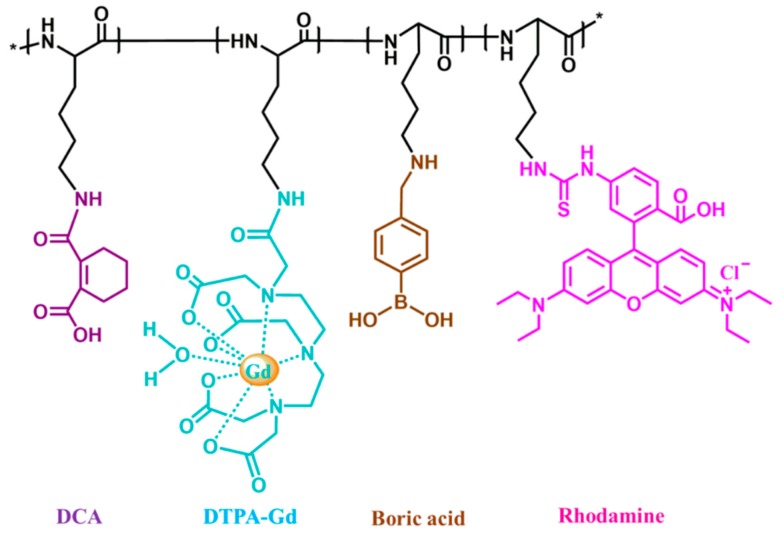
Schematic of tumor-targeting contrast agent PLL-B-DTPA-Gd-Rodamine-DCA. A series of functional groups, such as small molecule contrast agents (Gd^3+^), fluorescent dye (Rhodamine) and tumor-targeting group (boric acid groups) B were conjugated with the amine groups on the side chain of polylysine (PLL). The 3,4,5,6-tetrahydrophthalic anhydride (DCA) was also conjugated to neutralize the extra amine groups in PLL to make the whole agent exert negative charges.

Due to a large amount of amine groups in the side chain, polylysine was selected as the backbone of the contrast agent and was fully utilized to conjugate functional groups such as small molecule contrast agents (Gd^3+^), fluorescent dye (Rhodamine) and boric acid groups (B). We obtained two different B% content tumor-targeted MRI contrast agents. In addition, DCA was used to neutralize the extra amine groups in PLL to make the whole agent exert negative charges to avoid cytotoxicity while circulating in the blood. When reaching the tumor site, DCA hydrolyzed quickly in an acid environment and released the positive PLL-based contrast agent, thereby increasing the cancer cells’ uptake of themselves. The designed tumor-targeting contrast agent structure is shown in Figure 1. The ^1^H-NMR spectrum verification of every compound is given in the Supplementary Material.

**Figure 2:**
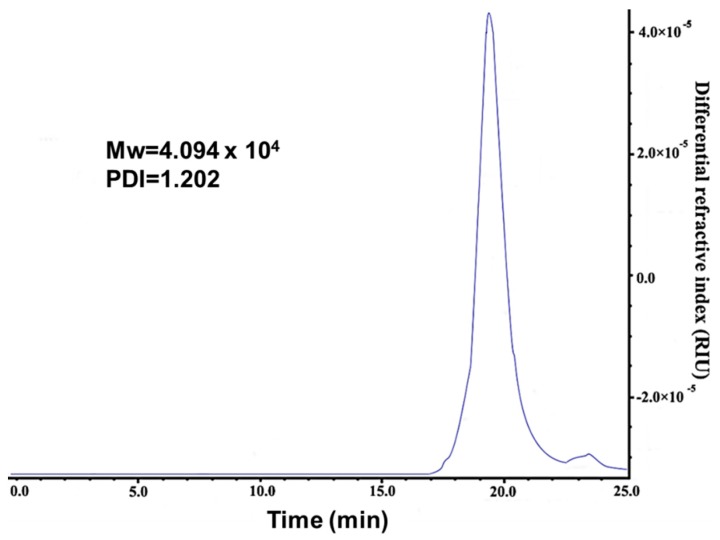
Molecular-weights of synthesized PLL measured by gel permeation chromatography (GPC) using dimethyl formamide (DMF) as the mobile phase.

The molecular weight of synthesized PLL was measured by GPC (gel permeation chromatography) using *N*,*N*-Dimethylformamide (DMF) as the mobile phase. It showed that PLL synthesized has a weight average molecular weight of 4 × 10^4^ with PDI 1.2, suggesting we have obtained a uniformly distributed PLL polymer (Figure 2).

To verify the content of small molecule contrast agent Gd^3+^ that had conjugated in PLL, inductively coupled plasma mass spectrometry (ICP-MS) was performed with both PLL-B(1%)-DTPA-Gd-Rodamine-DCA and PLL-B(5%)-DTPA-Gd-Rodamine-DCA. The Gd^3+^ content in PLL-B(1%)-DTPA-Gd-Rodamine-DCA was therefore calculated as 5.4%; the Gd^3+^ content in PLL-B(5%)-DTPA-Gd-Rodamine-DCA was 7.8%.

Furthermore, both PLL-B(1%)-DTPA-Gd-Rodamine-DCA and PLL-B(5%)-DTPA-Gd-Rodamine-DCA could form a water-soluble, clear and transparent solution if left for more than one month at room temperature, and no obvious aggregation could be seen during this period.

### 2.2. Charge Reversal Effect Confirmation

To confirm that DCA had successfully conjugated with PLL and can lead to a charge reversal effect, ζ-potential value was determined using laser Doppler anemometry for both DCA-conjugated PLL and unconjugated PLL (Table 1). This demonstrated that before conjugatation with DCA, the ζ-potential value of PLL-B(1%)-DTPA-Gd-Rodamine was 30.1 mV, and after DCA treatment it dropped to −16.5 mV. The same trend was found for PLL-B(5%)-DTPA-Gd-Rodamine: before DCA treatment the charge was about 31.7 mV, and after treatment it dropped to −28.7 mV, indicating that DCA can achieve a charge reversal effect, from positive to negative transition, when at physiology pH. Moreover, when incubating with pH 5.0 acid buffer for 1 h, the ζ-potentials of PLL-B(1%)-DTPA-Gd-Rodamine-DCA and PLL-B(5%)-DTPA-Gd-Rodamine-DCA were again reversed to positive charge 22.1 mV and 20.2 mV, respectively, suggesting that conjugated DCA hydrolyzed quickly in the acid environment and therefore led to breakage from the PLL side chains and charge reverse, which may increase the cell uptake in the tumor interstitium.

### 2.3. Longitudinal Relaxivity (R_1_) Measurement

**Figure 3:**
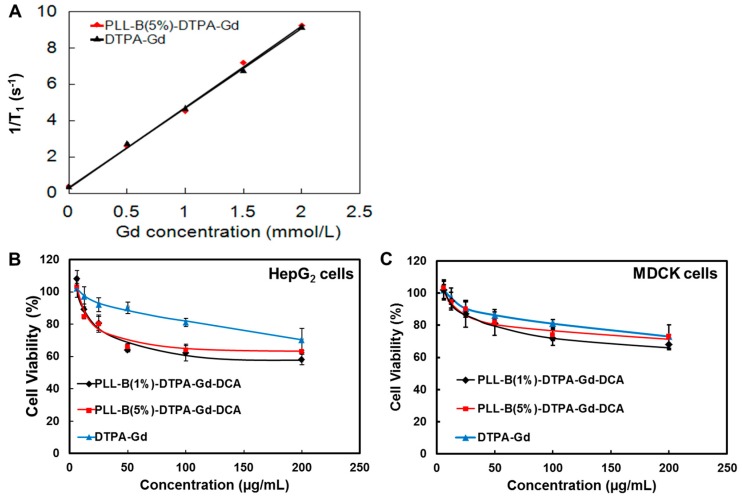
(**A**) Relaxation rates of PLL-B(5%)-DTPA-Gd and DTPA-Gd solutions at different concentrations (0.52 T and 32 °C). Cytotoxicity of PLL-B(1%)-DTPA-Gd-Rodamine-DCA and PLL-B(5%)-DTPA-Gd-Rodamine-DCA to HepG_2_ cells (**B**) and Madin–Darby Canine Kidney (MDCK) cells (**C**), estimated by MTT assay. Cells were cultured in DMEM medium for 24 h. All solid lines are fitted to the data.

To further investigate the imaging enhancement capability of the synthesized contrast agent, the T_1_ of PLL-B(5%)-DTPA-Gd solutions was determined on a 0.52-T MR system at 32 °C and the R_1_s were calculated from the slope of the curves of 1/T_1_ against the Gd^3+^ concentration shown in Figure 3A. The 1/T_1_ was linear with the Gd^3+^concentration and the R_1_ of DTPA-Gd (Magnevist^®^) was about 4.3 mM^−1^ s^−1^, which was similar to that reported in previous literature [24]. The relaxation rate (R_1_) of PLL-B(5%)-DTPA-Gd was about 4.5 mM^−1^ s^−1^, which was nearly the same as the small molecule contrast agent DTPA-Gd subjected to a magnetic field intensity of about 0.52 T at 32 °C. It was generally expected that macromolecular Gd chelate could hinder the water exchange between the coordinated H_2_O and bulk H_2_O around Gd chelate and therefore decrease R_1_s [25,26]. On the other hand, the conjugation of DTPA-Gd onto the rigid linear PLL polymer could increase the rigidity of the polymer and its rotational correlation time (_τ_ R) and therefore increase R_1_s [27]. Therefore, the novel contrast agent PLL-B(5%)-DTPA-Gd can exhibit comparable imaging enhancement to the DTPA-Gd (Magnevist^®^) used in clinic.

### 2.4. In Vitro MTT Assay

As PLL has been reported as maintaining some cytotoxicity in a panel of cell lines, due to the large amount of positive charge on the surface [28], the cytotoxicity of this novel PLL-based tumor-targeted magnetic resonance contrast agent was determined by MTT assay using HepG_2_ lung cancer cells as the model cancer cells and healthy dog kidney cells (Madin–Darby Canine Kidney (MDCK)) as the normal cells for comparing with small contrast agent DTPA-Gd. As Figure 3B shows, the cell proliferation ability was not significantly affected when drug concentration was around 20 μg/mL. As the drug concentration increased to 50 μg/mL, cancer cell growth was affected to some extent, but the viability rate was still above 65%. Even when the drug concentration increased to 200 μg/mL, cell viability rate could still maintain around 65%, which meant the majority of cells were still alive during incubation with PLL derivatives. Moreover, the cell viability of both PLL-B(1%)-DTPA-Gd-DCA and PLL-B(5%)-DTPA-Gd-DCA showed no significant difference in HepG_2_ cancer cells, indicating that both PLL-B(1%)-DTPA-Gd-DCA and PLL-B(5%)-DTPA-Gd-DCA had good biocompatibility at concentrations below 20 μg/mL. However, when the concentrations were increased to higher than 100 μg/mL, both agents showed cytotoxicity to some extent.

When treating normal MDCK cells, both agents showed less cytotoxicity than for cancer cells. This result may be attributed to the fact that PLL-B-DTPA-Gd-DCA with phenylboronic acid can more easily recognize the malignant cells, because sialic acid residues selectively highly overexpressed in the malignant cancer cells but not in the normal cells, and therefore accumulated more and led to higher cytotoxicity in cancer cells. Meanwhile, DTPA-Gd had also shown some but less cytotoxicity in HepG_2_ cells and MDCK cells. Even when the concentration reached 200 μg/mL, cell viability was still maintained at above 70%.

### 2.5. Tumor Cells Targeting Ability Measurement

**Figure 4:**
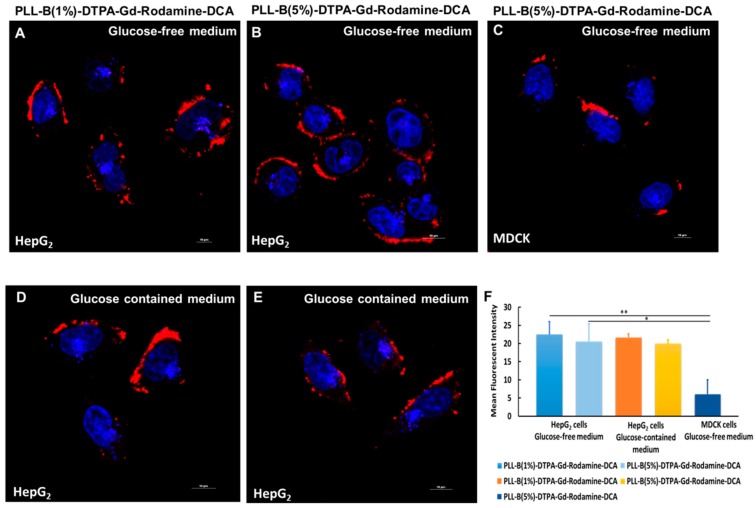
Intracellular colocalization of (**A**) PLL-B(1%)-DTPA-Gd-Rodamine-DCA on HepG_2_ cellsin glucose-free medium; (**B**) PLL-B(5%)-DTPA-Gd-Rodamine-DCA with cytomembrane on HepG_2_ cells in glucose-free medium; (**C**) PLL-B(5%)-DTPA-Gd-Rodamine-DCA on MDCK cells in glucose-free medium; (**D**) PLL-B(1%)-DTPA-Gd-Rodamine-DCA on HepG_2_ cells in glucose-containing medium; (**E**) PLL-B(5%)-DTPA-Gd-Rodamine-DCA on HepG_2_ cells in glucose-containing medium (Blue: DRAQ5; Red: PLL-B(1%)-DTPA-Gd-Rodamine-DCA/PLL-B(5%)-DTPA-Gd-Rodamine-DCA); (**F**) Image-based quantification of mean fluorescent intensity statistic using Image J software. Data represent the average fluorescent intensity of each group. * *p* < 0.05, ** *p* < 0.01.

It has been reported that sialic acid residues are highly overexpressed on the surface of malignant cancer cells and can easily be recognized by diol-phenylboronic acid (PBA) [29]. Therefore, the targeting ability of the PBA-based imaging contrast agent on both cancer cells and normal cells was determined by laser confocal microscope, as shown in Figure 4, and mean fluorescent intensity for each group was analyzed by Image J software. Compared to normal cells, PLL-B(1%)-DTPA-Gd-Rodamine-DCA and PLL-B(5%)-DTPA-Gd-Rodamine-DCA contrast agents were mostly distributed across the cancer cell HepG_2_ membrane, indicating that the boric acid groups of the polymers can specifically bind to the sialic acid residue’s structure on the cell membrane (Figure 4A,B) whereas on the normal cells, PLL-B(5%)-DTPA-Gd-Rodamine-DCA exhibited nearly no accumulation on the normal cells due to little sialic acid residues expressed in the normal cells (MDCK cells) (Figure 4C). The mean fluorescent intensity of binding ability showed significant difference between cancer cells and normal cells which strengthen the statement previously reported (Figure 4F).

Unfortunately, we did not observe any endocytosis, even when we incubated the PLL-B-DTPA-Gd-Rodamine-DCA with HepG_2_ cancer cells for 2 h. There may be two reasons for this: first, as PBA has a high affinity to sialic acid, the polymer that conjugated with PBA may initially recognize the sialic acid on the cell membrane and bind tightly with it, and therefore delay the time for endocytosis. Thus, the polymers may be internalized if the polymers are incubated for a longer time. Second, it has been reported that boric acid can recognize some glucose proteins, such as p-gp, on the cell membrane; therefore, the polymer may be efflux from the membrane after a certain period of incubation. Further study of modifying the polymers for enhancing endocytosis is still under way.

In addition, the target ability of 5% content of boronic acid contrast agent showed no significant difference to 1% boric acid content contrast agent, which may indicate that 1% content of boric acid has already reached the saturation concentration for boric acid targeting ability.

Furthermore, it is reported that boronic acids can bind to glucose, which is an abundant saccharide in the culture medium or blood. Thus, we tested whether glucose could lead to the suppression of polymers binding to tumor cells. We incubated both the PLL-B(1%)-DTPA-Gd-Rodamine-DCA and PLL-B(5%)-DTPA-Gd-Rodamine-DCA in either a glucose-free medium or a glucose-containing medium, and we found that there was nearly the same fluorescent intensity on the cell membrane in both medium, indicating that tumor-targeting ability was not affected by the glucose in the medium (Figure 4A vs. Figure 4D, Figure 4B vs. Figure 4E,F). The lower affinity of boronic acid to glucose than to Sia is due to the fact that d (+) glucose is present in the solution predominantly (>99%) in its pyranose anomeric form, which cannot combine with boronic acid.

In addition, Sakata et al. reported that phenylboronic acids were shown to be unable to bind to alpha-anomer of sialic acid, which is the dominant anomer, but bind to beta-anomer, which leads to the conclusion that PBA cannot selectively recognize the SA unit or discriminate between specific types of cell [30]. However, the structures of phenylboronate species also need to be taken into consideration. In our study, the complex showed positively charged when entering into the living cancer cells. The sialic acids are a family of C_9_ monosaccharides with a carboxyl group at the anomeric carbon atom (pKa = 2.2) that gives the molecule a negative charge at physiological pH. Therefore, it facilitated the recognition mechanism through electrostatic affinity to the negatively charged carboxylic groups of the Sia residues. Furthermore, the interaction between boric acid and SA may differ in the living cells because of the existence of serum and other proteins. Consequently, the PLL-B-DTPA-Gd-Rodamine-DCA derivatives shown in this study to have the ability to bind to the sialic acid residues structure on the cell membrane are reasonable.

## 3. Materials and Methods

### 3.1. Materials and Measurements

All the organic solvents were purchased from Sinopharm Chemical Reagent Co., Ltd. (Shanghai, China). Dimethyl sulfoxide was purified by distillation under vacuum over calcium hydride. Gadolinium chloride hexahydrate (GdCl_3_·6H_2_O) was bought from Alfa Aesar (Ward Hill, MA, USA). The cellulose ester dialysis membrane (molecular weight cut off, MWCO = 10,000) was purchased from Spectrum Laboratories Inc. (Rancho Dominguez, CA, USA). Amicon Ultra-15 centrifugal filter devices (MWCO = 10,000) were purchased from Millipore (Cork, Ireland).

The ^1^H-NMR spectra were recorded on a Bruker Avance DRX-400 NMR spectrometer (Bruker, Switzerland,) at room temperature. The sizes (hydrodynamic diameters) of the PCAs were measured using a dynamic light scattering instrument (Zetasizer Nano ZS, Malvern Instruments, Worcestershire, UK). Concentrations of Gd^3+^ solutions were measured by inductively coupled plasma mass spectrometry (ICP-MS, XSeries II, Thermo Scientific, Waltham, MA, USA). Gel permeation chromatography (GPC) was performed on a Wyatt GPC/SEC-MALS (Wyatt Technology Corporation, Santa Barbara, CA, USA) system equipped with a DAWN^®^ HELEOS^®^ II 18-angle static light scattering detector (Wyatt Technology Corporation, Santa Barbara, CA, USA) and an Optilab^®^ T-rEXTM refractive index detector (Wyatt Technology Corporation, Santa Barbara, CA, USA), and three columns (Wyatt Technology Corporation, Santa Barbara, CA, USA) in series (a MZ GPC-PRECOLUMN 50 × 8.0 mm MZ-Gel SD*plus* 100Å 10 μm, a 300 × 8.0 mm MZ-Gel SD*plus* 10E4Å 10 μm and a 300 × 8.0 mm MZ-Gel SD*plus* 100Å 10 μm) at 50 °C using DMF containing 50 mM LiBr as eluent at a flow rate of 0.80 mL min^−1^. Data were recorded and processed with ASTRA v6.0 (Wyatt Technology Corporation, Santa Barbara, USA) software.

The human hepatocellular carcinoma cell line HepG_2_ and the Madin–Darby Canine Kidney (MDCK) cell line were purchased from the China Center for Type Culture Collection (Wuhan, China).

### 3.2. Preparation and Processing

#### 3.2.1. Synthesis of PLL

**Figure 5:**
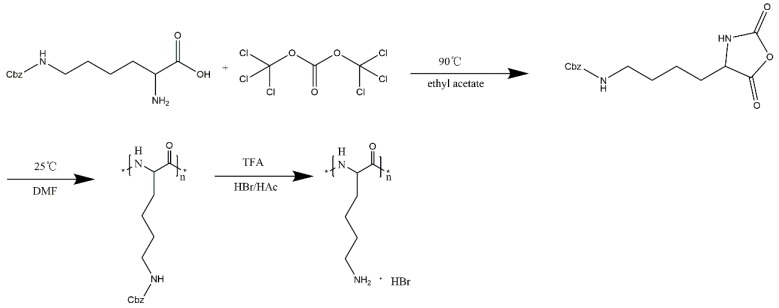
Synthesis route of PLL.

First, PLL was prepared using the methods reported in reference [31]. Details of the experimental procedures are as follows (Figure 5). The NCA derivatives were prepared by the method of Daly and Poche, with exceptions as noted below.

##### Preparation of NCA:

*N*-carboxyanhydrides that are isolated as oils should use no more than 1/3 of an equivalent of triphosgene and typically 10–20% less.

Ten grams of H-Lys(z)-OH were suspended in 150 mL of anhydrous ethyl acetate in a reaction flask fitted with a reflux condenser and N_2_ bubbler. After heating to reflux, 6.5 g of triphosgene were added at once and the reaction allowed to reflux under N_2_ for 4–5 h. Generally, the reaction became clear. The reaction was allowed to cool down to room temperature, which in some cases caused a solid (presumably the HCl salt of the starting amino acid) to precipitate. The reaction was then cooled in the stoppered reaction vessel to −5 °C.

The cold reaction was washed with 100 mL of de-ionized water, chilled to 0 °C. The ethyl acetate layer was then washed with 100 mL of 0.5% *w*/*v* NaHCO_3_, chilled to 0 °C. Anhydrous MgSO_4_ was used to dry the ethyl acetate layer. The clear solution was gravity filtered and concentrated to about 1/3 its original volume on a rotary evaporator below 30 °C. An equal volume of hexane or petroleum ether (30–60 °C cut) was then added to induce crystallization of the NCA. After chilling to −5 °C overnight, the NCA crystals were collected by suction filtration in a dry, N_2_ environment.

##### Polymerizations:

First, 9.2 g of NCA obtained from the last step were dissolved in 25 mL of dry DMF. One drop (<0.01 mL) of triethylamine was then added to the stirred solution. After stirring for three days, the polymer was dialyzed using an MW = 3500 dialysis bag to remove the DMF, and finally a white, lumpish solid (PLL-CBZ) was obtained.

##### PLL-CBZ deprotection:

The PLL-CBZ (8.7 g) was dissolved in 20 mL of trifluoroacetic acid (TFA) and 16 mL of HBr/HAc (33 wt %) was added dropwise, a bulk precipitate was gradually formed and stirred continuously at room temperature overnight. Five-fold volume excess of THF was added, centrifuged and repeated three times on the next day. The resulting white precipitate was dissolved in 50 mL of de-ionized water, placed in a MW = 3500 dialysis bag, and dialyzed for 24 h. The dialysate was taken out and lyophilized.

#### 3.2.2. Synthesis of Boric Acid Conjugated PLL (PLL-B)

**Figure 6:**
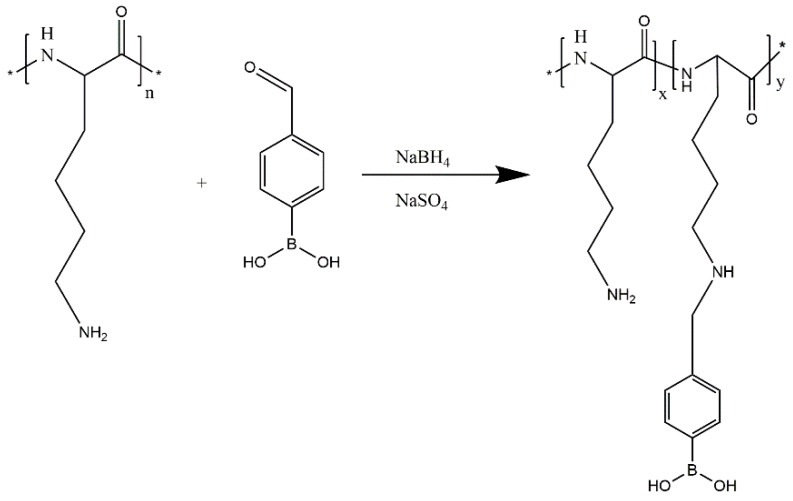
Synthesis route of PLL-B.

Synthesis route of PLL-B was as follows (Figure 6). Polylysine (0.2 g, 1.02 mmol NH_2_) was dissolved in 5 mL anhydrous methanol. Anhydrous sodium sulfate (0.5 g) and 4-formylbenzeneboronic acid (1.53 mg, 0.01 mmol NH_2_) were added and stirred at room temperature for 5 h. After the reaction, anhydrous sodium sulfate was removed by suction filtration, and sodium borohydride (1.16 mg, 0.0153 mmol of NH_2_) was added. The mixture was stirred continuously at room temperature for another 4 h. The anhydrous methanol was removed by rotary evaporation. The resulting solid was dissolved in 5 mL of water and then placed in a MW = 3500 dialysis bag and dialyzed for 24 h. The dialysate was taken out and lyophilized, and the obtained product was PLL-B (1%).

#### 3.2.3. Synthesis of PLL-B-DTPA

**Figure 7:**
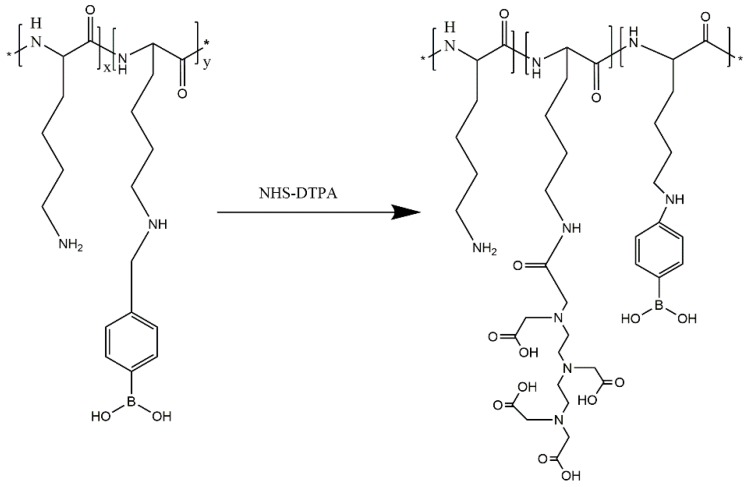
Synthesis route of PLL-B-DTPA.

The PLL-B-DTPA was prepared by the methods shown in Figure 7. The PLL-B (0.2 g, 1.01 mmol NH_2_) was dissolved in 5 mL of water, then DTPA-NHS (9.09 mmol NH_2_) was added and stirred at room temperature overnight. After the reaction was completed, the resulting solution was placed in a MW = 3500 dialysis bag and dialyzed for 24 h. The dialysate was taken out and lyophilized, and the obtained product was PLL-B(1%)-DTPA. The DTPA content is about 26% in terms of the integrated area.

#### 3.2.4. Synthesis of PLL-B-DTPA-Gd

The PLL-B(1%)-DTPA (0.505 mmol DTPA) was dissolved in 5 mL water. Then, 3 mL aqueous solution of cesium acetate (2 × 10^−4^ mol/mL, 1.2 eq) was added and stirred at room temperature for 2 h. After the reaction was completed, the resulting solution was placed in an ultrafiltration centrifuge tube and centrifuged at 4000 r/min for 30 min. The supernatant was taken out and lyophilized. The obtained product was PLL-B(1%)-DTPA-Gd.

#### 3.2.5. Tumor-Targeted Magnetic Resonance Contrast Agent Fluorescent Labelling

**Figure 8:**
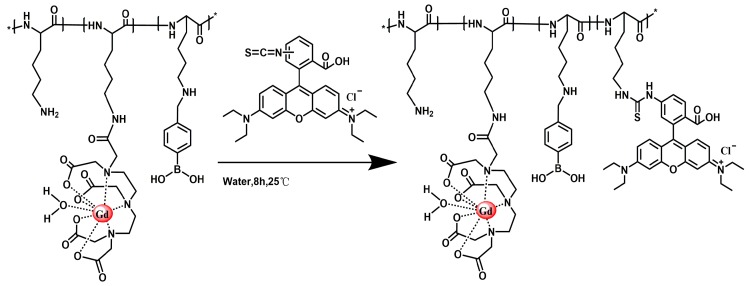
Synthesis route of PLL-B-DTPA-Rodamine.

Figure 8 shows the synthesis route of PLL-B-DTPA-Rodamine. The PLL-B(1%)-DTPA-Gd (0.1 g) was dissolved in 5 mL of water. Rhodamine isothiocyanate (1 mg) was added and stirred at room temperature for 8 h (Figure 8). After the reaction was completed, the resulting solution was placed in a MW = 3500 dialysis bag and dialyzed for 24 h and lyophilized. The resulting product was PLL-B(1%)-DTPA-Gd-Rodamine.

#### 3.2.6. Synthesis of PLL-B(1%)-DTPA-Gd-Rodamine-DCA

**Figure 9:**
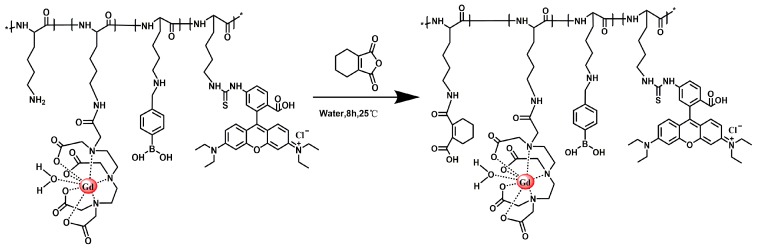
Synthesis route of PLL-B-DTPA-Rodamine-DCA.

The PLL-B(1%)-DTPA-Gd-Rodamine (0.5 g) was dissolved in 5 mL water. The DCA (1 g) was added and stirred at room temperature for 8 h (Figure 9). After the reaction was completed, the resulting solution was placed in a MW = 3500 dialysis bag and dialyzed for 24 h and lyophilized. The obtained product was PLL-B(1%)-DTPA-Gd- Rodamine-DCA.

### 3.3. Polymer Characterization

Concentrations of Gd^3+^ solutions were measured by inductively coupled plasma mass spectrometry (ICP-MS, XSeries II, Thermo Scientific, MA, USA).

The sizes (hydrodynamic diameters) of the PCAs were measured using a dynamic light scattering instrument (Zetasizer Nano ZS, Malvern Instruments, UK).

Gel permeation chromatography was performed on a Wyatt GPC/SEC-MALS (Wyatt Technology Corporation, Santa Barbara, USA) system equipped with a DAWN^®^ HELEOS^®^ II 18-angle static light scattering detector and an Optilab^®^ T-rEXTM refractive index detector, and three columns in series (a MZ GPC-PRECOLUMN 50 × 8.0 mm MZ-Gel SD*plus* 100Å 10 μm, a 300 × 8.0 mm MZ-Gel SD*plus* 10E4Å 10 μm and a 300 × 8.0 mm MZ-Gel SD*plus* 100Å 10 μm) at 50 °C using DMF containing 50 mM LiBr as eluent at a flow rate of 0.80 mL min^−1^. Data were recorded and processed with ASTRA v6.0 (Wyatt Technology Corporation, Santa Barbara, USA) software.

The ^1^H-NMR spectra were recorded on a Bruker Avance DRX-400 NMR spectrometer at room temperature.

### 3.4. Longitudinal Relaxivity (R_1_) Measurement

The longitudinal relaxation times (T_1_) were measured with a 0.52-T MicroMR Imaging and Analyzing System (Shanghai Niumag Corporation, Shanghai, China) at 32 °C. The T_1_ values of CA solutions with different Gd concentrations ranging from 0.5 to 2 mM in water were measured. The R_1_ values of CAs were defined by Formula (1).

(1/T_1_)_obs_=(1/T_1_)_d_ + R_1_ × [Gd] (1)

where [Gd] is the Gd concentration, (1/T_1_)_obs_ is the observed relaxation rate, and (1/T_1_)_d_ is the relaxation rate of water protons. The plot of (1/T_1_)_obs_ vs [Gd] gave R_1_ as its slope.

### 3.5. In Vitro MTT Assay

The cytotoxicity assay was carried out using the 3-(4,5-dimethylthiazol-2-yl)-2,5-diphenyltetrazolium bromide (MTT) cell proliferation kit (ATCC, Manassas, VA, USA). The HepG_2_ lung cancer cells were seeded in 96-well plates at an initial density of 4000 cells/well in 100 μL of DMEM medium and incubated for 24 h. The PLL-B(1%)-DTPA-Gd-DCA and PLL-B(5%)-DTPA-Gd-DCA in DMEM (100 μL) were added to the medium at different concentrations. Each concentration was replicated in 3 wells. Treated cells were incubated at 37 °C in humidified air with 5% CO_2_ for 48 h. The MTT solution (0.75 mg/mL) was then added to the wells for an additional 3-h incubation. Finally, 200 μL DMSO was added to each well, replacing the original medium to dissolve the formazan crystals. The absorbance in each well was determined at 562 nm using a microplate reader. The cell viability was calculated by Formula (2).

Relative cell viability (%) = Absorption test/Absorption control × 100%(2)

### 3.6. Tumor Cells Targeting Ability Measurement

The targeting effect of the drug on the cell membrane was observed by confocal microscope (Nikon A1, Kyushu, Japan). First, 1 mL of the medium which contains 1.5 × 10^5^ HepG_2_ cells or MDCK cells was inoculated into a confocal culture dish with a glucose-free medium or a glucose-containing medium. After 24 h incubation the medium was replaced, then PLL-B(1%)-DTPA-Gd-Rodamine-DCA and PLL-B(5%)-DTPA-Gd-Rodamine-DCA were added respectively. After incubation for 3 h, the medium was discarded and the lysosomal dye Lysotracker Green was added, culturing for another 2 h. Then, the lysosomal dye was washed away, the nuclear dye DRAQ5 was added and the medium was incubated for 15 min. Finally, the medium was drained and washed three times with 0.01 M PBS buffer. Then, the distribution of the drugs on the cells was observed under confocal microscope.

## 4. Conclusions

In summary, two PLL-B-DTPA-Gd-Rodamine-DCA derivatives with different contents of boric acid group as the tumor-targeting group on their surface were successfully prepared and characterized. The novel polymeric contrast agent showed a comparable contrast capability compared with DTPA-Gd used in clinic and good tumor targeting ability in vitro. Further in vivo contrasting performance is still under investigation. The moderate contrast properties, its facile synthesis and good biocompatibility, provide us with a new strategy for designing a novel contrast agent for early detection and diagnosis of tumors.

## Figures and Tables

**Table 1:** ζ-potential of PLL-B-DTPA-Gd-Rodamine and PLL-B-DTPA-Gd-Rodamine-DCA in different conditions.

	PLL-B(1%)-DTPA-Gd-Rodamine in pH 7.4	PLL-B(1%)-DTPA-Gd-Rodamine-DCA in pH 7.4	PLL-B(1%)-DTPA-Gd-Rodamine-DCA in pH 5.0
ζ-potential	30.1 mV	−16.5 mV	22.1 mV
	PLL-B(5%)-DTPA-Gd-Rodamine in pH 7.4	PLL-B(5%)-DTPA-Gd-Rodamine-DCA in pH 7.4	PLL-B(5%)-DTPA-Gd-Rodamine-DCA in pH 5.0
ζ-potential	31.7 mV	−28.8 mV	20.2 mV

## Supplementary Materials

The following are available online. Figure S1: ^1^H-NMR spectra of PLL in D_2_O, Figure S2: ^1^H-NMR spectra of PLL-B (1%) in D_2_O, Figure S3: ^1^H-NMR spectra of PLL-B (5%) in D_2_O, Figure S4: ^1^H-NMR spectra of PLL-B(1%)-DTPA in D_2_O, Figure S5: ^1^H-NMR spectra of PLL-B(5%)-DTPA in D_2_O.
