# Supplementary material for: Preparation and Properties of Tumor-Targeting MRI Contrast Agent Based on Linear Polylysine Derivatives
Source: Molecules. 2019 Apr 15;24(8):1477. doi: 10.3390/molecules24081477 (PMC6515188; doi:10.3390/molecules24081477)
Supplement: Supplementary file 1 [file molecules-24-01477-s001.pdf]

1 Preparation and Properties of Tumor-Targeting MRI Contrast Agent Based on Linear Polylysine Derivatives PLL ^{1}H NMR Spectrum Verification

^{1}H NMR (400 MHz, D_2O): 4.2-4.3 ppm (s, H, -COCHNH-), 2.9-3.0 ppm (d, 2H, -$CH_2CH_2NH_2$), 1.3-1.8 ppm (d, 6H, -$CHCH_2CH_2CH_2CH_2$-)

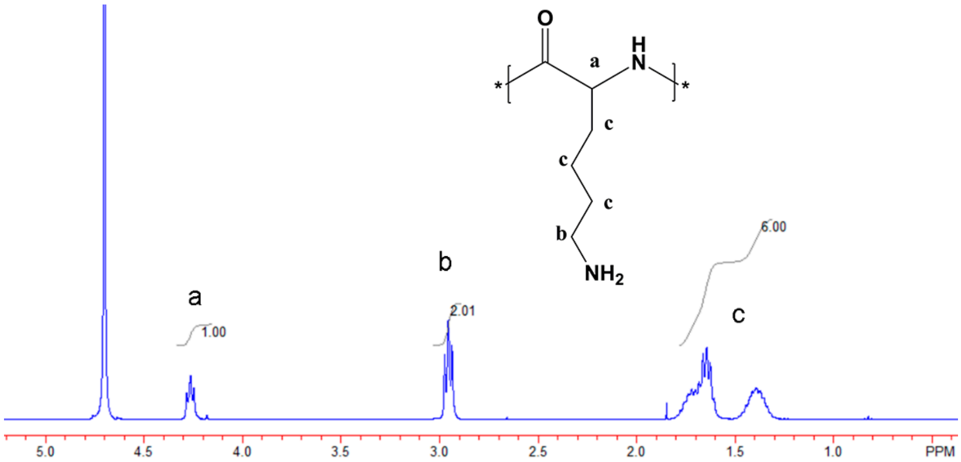

Figure S1. ^{1}H NMR spectra of PLL in D_2O

2 Boric Acid Conjugated PLL (PLL-B) ^{1}H NMR Spectrum Verification

PLL-B(1%): ^{1}H NMR (400 MHz, D_2O): 4.2-4.3 ppm (s, H, -COCHNH-), 2.6-2.8 ppm (d, 2H, -$CH_2CH_2NH_2$，-$NHCH_2C$-), 1.3-1.8 ppm (d, 6H, -$CHCH_2CH_2CH_2CH_2$-). The boric acid content is about 1% in terms of the integrated area.

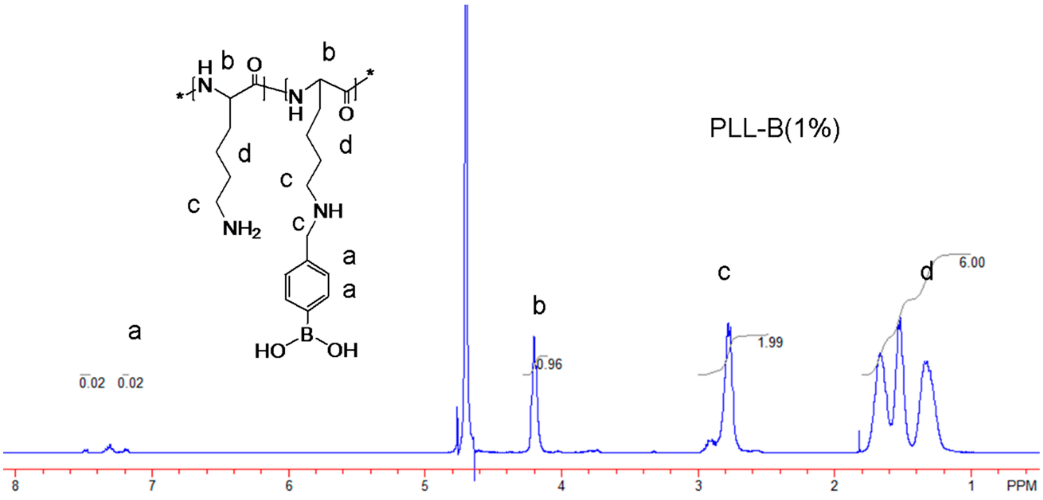

Figure S2. ^1H NMR spectra of PLL-B(1%) in D_2O

PLL-B(5%): ^1H NMR (400 MHz, D_2O): 4.2-4.3 ppm (s, H, -COCHNH-), 2.5-2.8 ppm (d, 2H, $-CH_2CH_2NH_2$，$-NHCH_2C-$), 1.3-1.8 ppm (d, 6H, $-CHCH_2CH_2CH_2CH_2-$). The boric acid content is about 5% in terms of the integrated area.

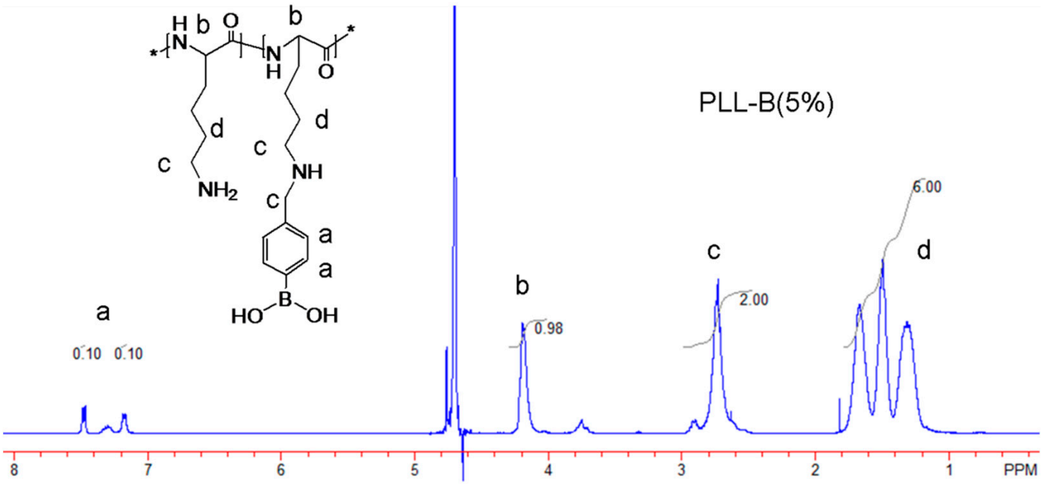

Figure S3. ^1H NMR spectra of PLL-B(5%) in D_2O

3 PLL-B (1%)-DTPA ^1H NMR Spectrum Verification

^1H NMR (400 MHz, D_2O): 4.2-4.3 ppm (s, H, -COCHNH-), 3.8 ppm (d, 2H, -$CH_2CH_2NH_2$，$-NHCH_2C-$), 1.3-1.8 ppm (d, 6H, $-CHCH_2CH_2CH_2CH_2-$), 2.8-3.5 ppm (DTPA), 7.2-7.6 ppm (4H, benzene). The DTPA content is about 26% in terms of the integrated area.

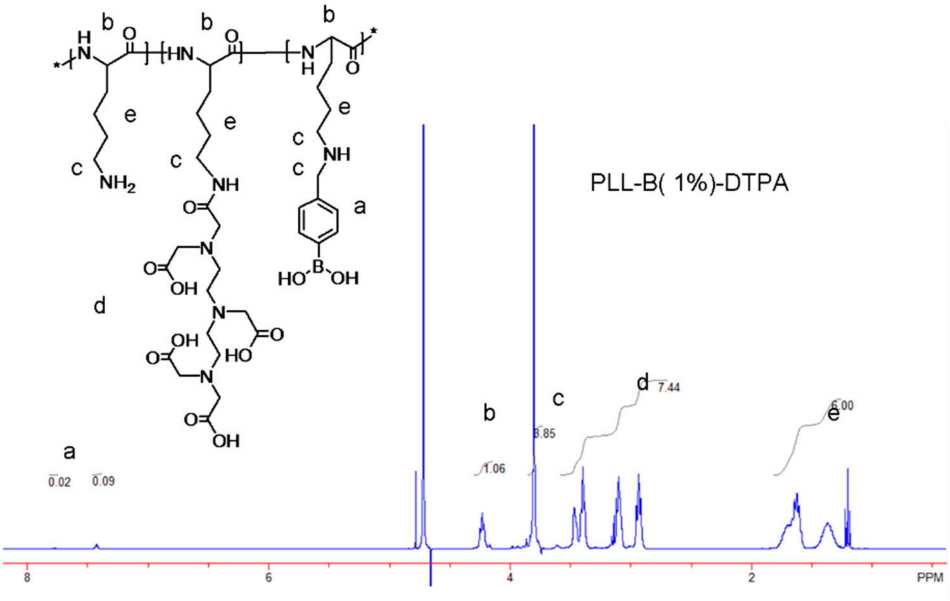

Figure S4. ^{1}H NMR spectra of PLL-B(1%)-DTPA in D_2O

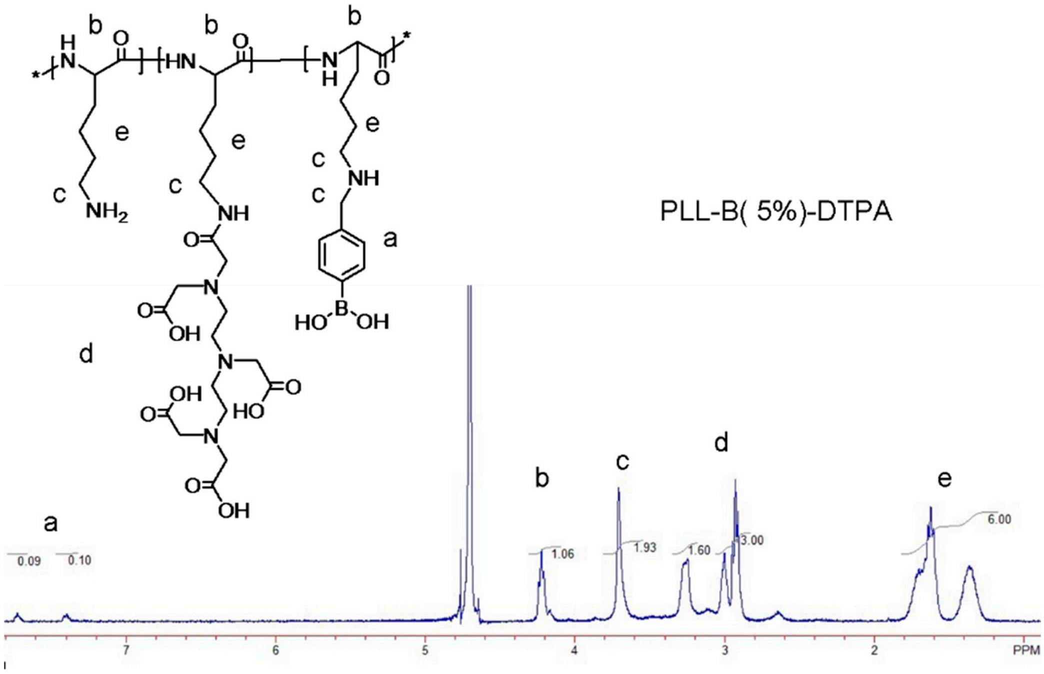

Figure S5. ^{1}H NMR spectra of PLL-B(5%)-DTPA in D_2O

4 PLL-B (1%)-DTPA-DCA ^{1}H NMR Spectrum Verification in pH7.4 and pH5.0

^{1}H NMR (400 MHz, D_2O): 4.2-4.3 ppm (s, H, -COCHNH-), 3.8 ppm (d, 2H, -$CH_2CH_2NH_2$，-$NHCH_2C$-), 1.3-1.8 ppm (d, 6H, -$CHCH_2CH_2CH_2CH_2$-), 1.8-2.4 ppm (DCA), 2.8-3.5 ppm (DTPA), 7.2-7.6 ppm (4H, benzene).

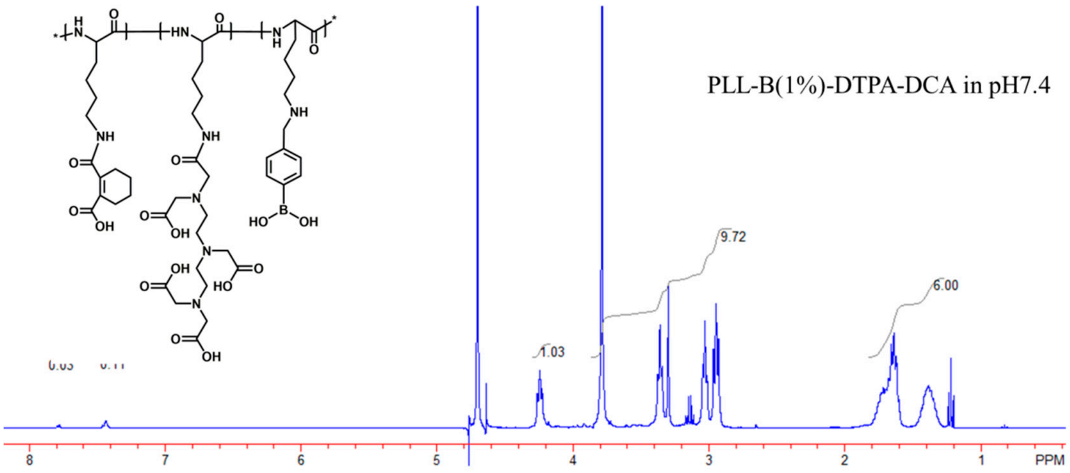

Figure S6. ^{1}H NMR spectra of PLL-B(1%)-DTPA-DCA in D_2O in pH7.4

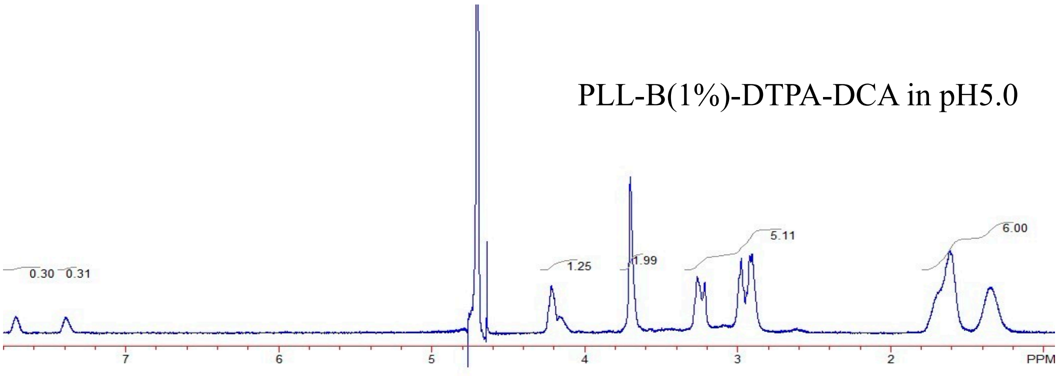

Figure S7. ^{1}H NMR spectra of PLL-B(1%)-DTPA-DCA in D_2O in pH5.0
